# A Dynamical Bifurcation Model of Bipolar Disorder Based on Learned Expectation and Asymmetry in Mood Sensitivity

**DOI:** 10.1162/cpsy_a_00021

**Published:** 2018-12

**Authors:** Shyr-Shea Chang, Tom Chou

**Affiliations:** Department of Mathematics, University of California, Los Angeles, Los Angeles, California, USA; 2 Department of Mathematics, University of California, Los Angeles, Los Angeles, California, USA; 3 Department of Biomathematics, University of California, Los Angeles, Los Angeles, California, USA

**Keywords:** bipolar disorder, behavioral approach system, dynamics, affective bias, prediction error

## Abstract

Bipolar disorder is a common psychiatric dysfunction characterized by recurring episodes of mania and depression. Despite its prevalence, the causes and mechanisms of bipolar disorder remain largely unknown. Recently, theories focusing on the interaction between emotion and behavior, including those based on dysregulation of the so-called behavioral approach system (BAS), have gained popularity. Mathematical models built on this principle predict bistability in mood and do not invoke intrinsic biological rhythms that may arise from interactions between mood and expectation. Here we develop and analyze a model with clinically meaningful and modifiable parameters that incorporates the interaction between mood and expectation. Our nonlinear model exhibits a transition to limit cycle behavior when a mood-sensitivity parameter exceeds a threshold value, signaling a transition to a bipolar state. The model also predicts that asymmetry in response to positive and negative events can induce unipolar depression/mania, consistent with clinical observations. We analyze the model with asymmetric mood sensitivities and show that large unidirectional mood sensitivity can lead to bipolar disorder. Finally, we show how observed effects of lithium- and antidepressant-induced mania can be explained within the framework of our proposed model.

## INTRODUCTION

Bipolar disorder is characterized by cycling between manic and depressive episodes (Geller & Luby, 1997). Its prevalence is estimated to be 0.3%–1.5% of the total population (Weissman et al., 1996). The lifetime cost for a single patient can reach several million US dollars (Begley et al., 2001), and medication use associated with bipolar disorder comprises about 7% of that used to treat all mental disorders (Whiteford et al., 2013). Bipolar disorder has a serious societal impact, with 65.5 workdays lost per year per patient (Kessler et al., 2006) and its early onset a major risk factor for suicide (Hawton, Sutton, Haw, Sinclair, & Harriss, 2005). Despite the significance of bipolar disorder, there is limited structural understanding of the underlying mechanisms (Geddes & Miklowitz, 2013). Modern techniques, such as functional magnetic resonance imaging (fMRI), have located neural circuits, including limbic networks and attentional systems, whose dysfunction may be correlated with bipolar disorder (Chen, Suckling, Lennox, Ooi, & Bullmore, 2011; Strakowski, Adler, Holland, Mills, & DelBello, 2004). However, how the dysfunction of these circuits leads to emotional vulnerability remains unclear.

To understand the mechanism of bipolar disorder and accelerate the development of treatment (Geddes & Miklowitz, 2013), many mathematical models have been proposed and fit to experimental data. An oscillation in mood, either observed or self-reported, is the defining feature of bipolar disorder (Geller & Luby, 1997). Thus early models focus on explaining this oscillation (Bonsall, Geddes, Goodwin, & Holmes, 2015; Daugherty et al., 2009; Goldbeter, 2011). The models describe mood as being formed from an intrinsically oscillatory brain circuit and explain self-reported mood scores as well as the effects of medication. Following these studies, a natural next step is to clarify the mechanism of the oscillations and distinguish key differences between normal individuals and patients with bipolar disorder (see also Goldbeter, 2011). A popular theory states that dysregulation of the behavioral approach system (BAS) and the resulting interaction between mood, expectation, and behavior can explain bipolar disorder (Urošević, Abramson, Harmon-Jones, & Alloy, 2008). Psychological observations provide evidence of malfunction of the BAS, and models have been built to explain bipolar disorder based on this malfunction (Steinacher & Wright, 2013). A key difference between BAS-based models and some earlier models is that BAS models show bistability in mood instead of oscillations and require external input or noise to trigger switching between states of mania and depression (Cochran, Schultz, McInnis, & Forger, 2017).

Can a model exhibiting periodic mood oscillations and other observed features be derived from self-contained models that incorporate expectation and behavior? Recent psychological experiments have shown that emotion is affected by the mismatch between expectation and reality instead of the reward value (Rutledge, Skandali, Dayan, & Dolan, 2014). Theoretically, it has been shown that the interaction between mood and expectation captures the qualitative features of self-reported mood in psychological experiments (Eldar & Niv, 2015) and can indeed lead to bipolar disorder. In reality, there are many factors that can interact with mood and expectation. For example, it has been suggested (Eldar, Rutledge, Dolan, & Niv, 2016; Mason, Eldar, & Rutledge, 2017) and reported both clinically and in psychological experiments (Gotlib, Krasnoperova, Yue, & Joormann, 2004; Pulcu & Browning, 2017) that the sensitivities toward positive and negative events can be different.

In this work, we develop and analyze a variant of the models proposed by Eldar & Niv (2015), Eldar et al. (2016), and Mason et al. (2017). Like these models, our model is based on an interaction between mood and expectation and incorporates experimentally measurable variables (e.g., see Pulcu and Browning, 2017). Following a similar analysis as that in Eldar and Niv (2015), we prove that our model exhibits oscillatory mood behavior when a particular psychological parameter, the mood sensitivity, exceeds a threshold value. Our analysis further quantifies the amplitude and frequency of oscillations in mood and expectation. We also explore the effects of different amplitudes of responses of mood to positive and negative events—or asymmetric mood sensitivity. In our model, we will show that depending on the initial level of asymmetry, changing the response to either positive or negative events may lead to a bipolar state. We introduce a randomly fluctuating reality and show that it preserves many of the qualitative features predicted under constant reality but produces irregular mood trajectories that qualitatively resemble observations (Bonsall et al., 2015). Finally, we model the effects of pharmaceutical intervention, including those of antidepressants and lithium.

## MATHEMATICAL MODEL

We propose a continuous-time model based on interactions between the dynamical variables of mood *m*(*t*), expectation *v*(*t*), and reality *r*(*t*):

$$\frac{\text{d}m}{\text{d}t}=\eta_{m}(fm+r-v)-km-k_{3}m^{3}$$ (1)

$$\frac{\text{d}v}{\text{d}t}=\eta_{v}(fm+r-v).$$ (2)

Here, *η*_*m*_ and *η*_*v*_ are learning rates for mood and expectation, respectively; *f* is a scale factor for how mood contributes to *perceived* reality *fm* + *r*; and *k*, *k*_3_ are linear and cubic recovery rates for mood, respectively. The perceived reality *fm* + *r* in our model represents, in a linear way, the modification that mood has on reality. Thus *fm* + *r* − *v* reflects the extent to which an individual is surprised and how strongly she should respond. Unlike for the expectation *v*(*t*), the mood equation contains a separate term that drives it to a baseline level, even after positive reality events, such as winning a lottery (Brickman, Coates, & Janoff-Bulman, 1978). This recovery “force” for the mood is captured by the −*km*(*t*) term, with *k*^−1^ a mood relaxation time scale. We will see that this linear recovery term is essential for explaining the cyclothymic transition from normal to bipolar models. Finally, if mood is viewed as a physiological quantity, its magnitude should be bounded. To prevent the mood from growing indefinitely, we include a higher order nonlinear cubic term (corresponding to a quartic “potential”) in the mood equation. Thus both linear and cubic recovery terms play key roles in explaining how the bipolar disorder occurs in our model.

The reality *r*(*t*) is derived from external events and is not affected by personal mood or expectation. This assumption distinguishes the proposed model from those based on the BAS (Steinacher & Wright, 2013). By eliminating the expectation *v*(*t*), our model can also be written in terms of a single nonlinear oscillator in mood [assuming that *r*(*t*) is differentiable]:

$$\frac{{\text{d}}^{2}m}{\text{d}t^{2}}-(f\eta_{m}-k-\eta_{v}-3k_{3}m^{2})\frac{\text{d}m}{\text{d}t}+\eta_{v}km+\eta_{v}k_{3}m^{3}=\eta_{m}\frac{\text{d}r}{\text{d}t}.$$ (3)

This is a Liénard equation (Strogatz, 2014) similar to the van der Pol oscillator invoked in previous theories (Bonsall et al., 2015; Daugherty et al., 2009). The main new features here are the forcing term *η*_*m*_(d*r*/d*t*) that depends on *changes* in reality, the higher order term *η*_*v*_*k*_3_*m*^3^, and a possibly nonconstant parameter *η*_*m*_, as we will explore later in this section. The main mechanism behind our model is that positive and negative surprises, that is, the difference between perceived reality and expectation, drive mood in corresponding directions, which in turn adjusts the perceived reality and speeds up the adaptation of expectation. In this sense, the rate of change of mood is analogous to the momentum of a damped harmonic oscillator (Eldar et al., 2016). From our daily experience, it is apparent that mood changes the way we perceive reality: A minor drawback may have no effect on us when we are happy but can be a source of depression if we are not in good spirits.

Our model is actually a variant of the one proposed in Eldar et al. (2016) but differs in three ways. First, the mood affects the perceived reality in the mood dynamics. Second, the linear decay term −*km* has an independent parameter *k*. This is different from the Eldar et al. (2016) model, in which the mood recovery rate is assumed to be the same as the mood learning rate *η*_*m*_ and allows for more mathematical generality, since psychologically, the mood recovery rate may be able to vary independently from the mood learning rate. Finally, as noted earlier, we have added a cubic mood recovery term −*k*_3_*m*^3^. This cubic suppression term and the linear decay term are essential for the system to admit limit cycle behavior that captures bipolar disorder.

The model exploits a similar central mechanism as that proposed in Eldar and Niv (2015) but with a number of technical differences. In Eldar and Niv (2015), the mood is defined through a sigmoidal tanh function of a quantity that reflects recent “prediction-error history.” In contrast, the mood in our model directly reflects the prediction-error history but is susceptible to the effects of a higher order recovery term −*k*_3_*m*^3^, distinguishing it from both Eldar and Niv (2015) and Eldar et al. (2016). This difference represents two mechanisms for bounding the mood: explicitly specifying the limits of the mood through the tanh function and limiting the mood through a general (allowed by symmetry) cubic “force” term in the dynamics. Our model is also different from Eldar and Niv (2015) in that the effect of mood on perceived reality assumes an additive rather than a multiplicative form. In summary, our model has a simpler mathematical form yet generalizes the previous models by Eldar and Niv (2015), Eldar et al. (2016), and Mason et al. (2017) in a way that allows for a clean, self-contained mathematical analysis and a spectrum of qualitative behaviors.

Throughout this article, we will explore the effects of two forms of the reality function *r*(*t*): a constant *r*(*t*) = *r*_0_ and a random *r*(*t*). In the random case, we assume a piecewise constant form for *r*(*t*) with normally distributed values and log-normally distributed times between jumps. This functional form reflects the abrupt nature of changes in reality such as salary raises or the death of relatives that cause a dramatic change lasting for certain periods of time. We set the mean and standard deviation of *r*(*t*) to be 0, *σ*_*r*_. The time intervals between jumps in *r*(*t*) are drawn from a log-normal distribution with mean log time 1/*k*_*r*_ and standard deviation of the log time 1/*k*_*r*_. The parameters *f*, *k*, *k*_3_, *η*_*v*_ are treated as positive constants throughout the article. It has also been shown that learning rates *η*_*m*_ can be different for positive and negative events (Pulcu & Browning, 2017), which we model using a Heaviside function of *fm* + *r* − *v*:

$$\eta_{m}=\left\{\begin{array}{ll} \eta_{m}^{+} & \;\text{if}\;fm+r-v>0\\ \eta_{m}^{-} & \;\text{if}\;fm+r-v\leq 0, \end{array}\right.$$ (4)

where *η*_*m*_^+^, *η*_*m*_^−^ are positive constants. We will show in the Results section how asymmetry in *η*_*m*_ (the case *η*_*m*_^+^ ≠ *η*_*m*_^−^) can influence the onset of disorders. The parameters are tuned such that the time scale of mood variation matches the experimental data in Bonsall et al. (2015), except in Figure 3, the time scale is tuned so that the adaptation of mood to positive and negative events agree with common experience.

To better connect our results with clinical observations, we calculate (QIDS-SR16) Quick Inventory of Depressive Symptomatology scores (Rush et al., 2006) from our model. The QIDS-SR16 (QIDS for short) is commonly used for analyzing and testing treatments of bipolar disorder (Bonsall, Wallace-Hadrill, Geddes, Goodwin, & Holmes, 2012; Holmes et al., 2016) and consists of a 16-item self-test that measures the level of depression. We calculate this score by taking −min(0, *m*) since negative mood corresponds to depression. In principle, our model predicts mood and expectation but does not capture all specific indicators of depression and mania. Here we adjust the scale of mood to QIDS score to connect our work with clinical observations. The system (1, 2) is solved by explicit fourth to fifth order Runge–Kutta solvers (Dormand & Prince, 1980), carried out by the ode45 function in MATLAB.

## RESULTS

### Mood and Expectation Become More Oscillatory as the Mood Sensitivity Increases

For normal subjects, we expect that if the reality *r*(*t*) = *r*_0_ is constant, the expectation should approach *r*_0_ and the mood will relax to zero as there are no additional stimuli; this justifies shifting *r*_0_ → 0 without loss of generality and linearizing Equations 1–2 around the fixed point (*m*, *v*) = (0, 0). In this way, we can define the parameter regime within which the origin becomes linearly unstable (suggesting the onset of bipolar disorder) and which parameters are crucial for stability. Before we study more general cases, we first assume symmetry in the mood learning rate *η*_*m*_, that is, *η*_*m*_^+^ = *η*_*m*_^−^, to gain insight into the basic model.

Upon linearizing Equations 1–2 or Equation 3 about (*m*, *v*) = (0, 0) for *r* = 0, we find the two eigenvalues

$$\lambda_{\pm }=\frac{\eta_{m}f-\eta_{v}-k}{2}\pm \frac{\sqrt{\Delta }}{2},$$ (5)

where the discriminant

$$\Delta \equiv (\eta_{m}f-\eta_{v}-k{)}^{2}-4\eta_{v}k.$$ (6)

Thus, the origin is linearly stable when *fη*_*m*_ − *k* − *η*_*v*_ < 0 and Re(*λ*_±_) < 0 and unstable when *fη*_*m*_ − *k* − *η*_*v*_ > 0, corresponding to at least one eigenvalue containing a positive real part. This analysis agrees with that of Eldar et al. (2016), in which bipolar disorder arises either when *f* is large or *η*_*m*_ ≫ *η*_*v*_. Here we focus on mood and base our study on the quantity *fη*_*m*_, which we call the mood sensitivity parameter. In the linearly stable case, the system can support transiently oscillating behavior in mood and expectation, similar to that of a damped harmonic oscillator (Marion, 2013). For oscillatory behavior that is underdamped or undamped, there are potentially associated qualitative clinical presentations, such as cyclothymic and bipolar personalities (Mason et al., 2017).

**Figure 1.**
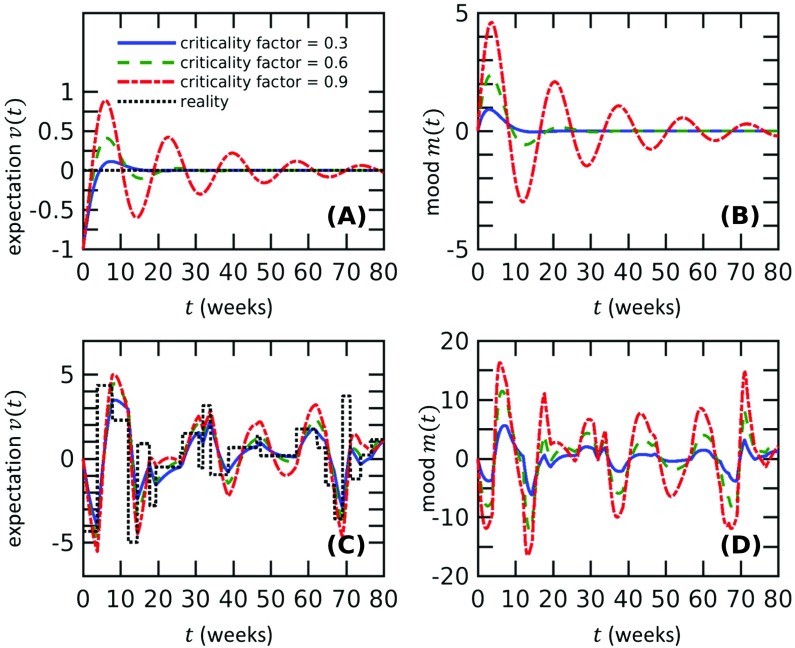
**The mood and expectation of normal subjects become more oscillatory as the mood sensitivity *fη*_*m*_ increases toward the critical value *η*_*v*_ + *k* from below.** A) Oscillations in expectation are highly damped for normal subjects (blue solid, *fη*_*m*_ = 0.3(*η*_*v*_ + *k*)) but become less damped when the mood sensitivity increases (green dotted, *fη*_*m*_ = 0.6(*η*_*v*_ + *k*), and red dash-dotted, *fη*_*m*_ = 0.9(*η*_*v*_ + *k*)). Since we start the solutions at (*m*, *v*) = (0, −1), the constant reality *r*(*t* > 0) = 0 represents a permanent increase in reality from *r*(*t* < 0) = −1. The numerical values *η*_*v*_ = 0.37, *f* = 0.3, *k* = 0.37, and *k*_3_ = 2.8 × 10^−3^ are used in all subfigures. B) The mood shows similar oscillatory behavior that becomes less damped with increasing mood sensitivity. C) When subjected to random reality events, models with large mood sensitivities exhibit larger responses in expectation. D) Similarly, the fluctuation in mood is greater in systems with larger mood sensitivity under random reality conditions. Realizations of the random reality function are generated as described in the Mathematical Model section, with *σ*_*r*_ = 2, *k*_*r*_ = 1. In C) and D), mood and expectation are initialized at (*m*, *v*) = (0, 0).

Oscillation frequencies are characterized by the imaginary part of the eigenvalues, determined by the sign of Δ. When Δ is positive, there will be no oscillation in the solutions, while negative Δ corresponds to oscillatory solutions, with oscillation frequency determined by $\sqrt{\left|\Delta \right|}/2$. As a function of the mood sensitivity parameter *fη*_*m*_, we see that Δ is a parabola with minimum at *fη*_*m*_ = *η*_*v*_ + *k*, the critical value for linear stability, with a negative discriminant −4*η*_*v*_*k*. Thus, as *fη*_*m*_ increases toward the critical value *η*_*v*_ + *k*, the mood and expectation become oscillatory with the frequency in the oscillations increasing. As the mood sensitivity *fη*_*m*_ exceeds the critical value, a Hopf bifurcation occurs, the linearized dynamics become unstable, and linear analysis can no longer predict system behavior. This argument suggests that mood fluctuations even in normal (subthreshold) systems increase as the mood sensitivity increases. We verify these arguments by numerically solving Equations 1 and 2 using constant *r*(*t*) = 0 and different mood sensitivities. The numerical solutions show that the oscillation frequency in mood and expectation increases as the mood sensitivity *fη*_*m*_ becomes larger, as predicted by our linear analysis (Figures 1A and 1B). Notice that when *k* = 0 and there is no linear dissipation of mood, the eigenvalues are strictly real and the system does not support a cyclothymic regime across the stability threshold. The mood dynamics transition from exponentially decaying directly to exponentially growing behavior.

Linear stability analysis does not fully apply when the reality *r*(*t*) is time dependent. However, numerical solutions show that for larger *fη*_*m*_, the expectation *v*(*t*) deviates more from reality *r*(*t*) and the mood *m*(*t*) experiences higher variations about its baseline (Figures 1C and 1D). These results suggest that the mood sensitivity controls a spectrum of personality responses, from normal to cyclothymic, and is a key determinant in triggering bipolar disorder as its threshold is exceeded.

### A Limit Cycle Occurs as Mood Sensitivity Crosses the Critical Value, Representing a Bipolar State

Once the mood sensitivity *fη*_*m*_ exceeds the threshold *η*_*v*_ + *k*, linear analysis no longer holds since the origin becomes unstable and nonlinearities quickly become important. However, for two-dimensional systems, we can rely on the Poincaré–Bendixson theorem to predict the existence of a limit cycle, a periodic solution that attracts solutions starting nearby (Strogatz, 2014). For this analysis, and in the rest of this subsection, we still assume *η*_*m*_^+^ = *η*_*m*_^−^ and a constant *r*(*t*) = 0. Since the origin is linearly unstable, we search for a limit cycle by constructing an outer boundary on which the vector fields are pointing inward. One way of finding this boundary is to draw a rectangle whose edges connect two nullclines $v=(f-\frac{k}{\eta_{m}})m-\frac{k_{3}}{\eta_{m}}m^{3}$ and *v* = *fm*. Since *η*_*m*_^+^ = *η*_*m*_^−^, both nullclines are rotationally symmetric, allowing us to find the distance to the right edge of the boundary by setting −fm* equal to the *m*-nullcline:

$$-fm^{*}=(f-\frac{k}{\eta_{m}})m^{*}-\frac{k_{3}}{\eta_{m}}m^{*3},$$ (7)

which leads to

$$m^{*}=\sqrt{\frac{2f\eta_{m}-k}{k_{3}}}.$$ (8)

**Figure 2.**
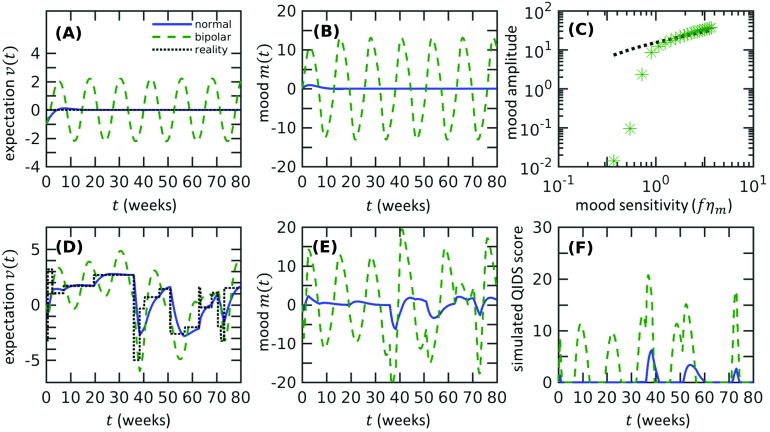
**Our theory predicts that the onset of bipolar disorder occurs through a supercritical Hopf bifurcation as the mood sensitivity *fη*_*m*_ crosses the threshold value *η*_*v*_ + *k* and a limit cycle in mood *m*(*t*) is established.** A) In a bipolar state, the expectation *v*(*t*) (dotted green) persistently oscillates, in contrast to the normal case (solid blue). We set the reality *r*(*t* > 0) = 0 and use (*m*, *v*) = (0, −1) as the initial condition. The bipolar state is modeled using *fη*_*m*_ = 1.5(*η*_*v*_ + *k*), whereas the normal state is computed using *fη*_*m*_ = 0.3(*η*_*v*_ + *k*). The numerical values *η*_*v*_ = 0.37, *f* = 0.3, *k* = 0.37, and *k*_3_ = 2.8 × 10^−3^ are used in all subfigures. B) Mood of bipolar subjects also persistently oscillates. C) The magnitude of mood oscillations increases as the mood sensitivity *fη*_*m*_ increases. The amplitude of oscillations obtained from numerical simulations (green stars) compares well to amplitude estimates using Equation 8 (black dots) when *fη*_*m*_ ≫ *η*_*v*_ + *k*. The estimates from Equation 8 are normalized by the mean of calculated amplitudes to emphasize the match in the power law. D) Expectation *v*(*t*) in the bipolar state responds to changes in reality but remains oscillatory (green dashed). This behavior is distinct from the expectation of normal subjects (solid blue curve) that more closely follows the reality function. E) Under the same reality function as in D), the mood is much more oscillatory in the bipolar state (green dashed curve) than in the normal state (solid blue curve). F) The model predicts intermittent spikes in the QIDS score. Realizations of the reality function are generated as described in the Mathematical Model section, with *σ*_*r*_ = 2, *k*_*r*_ = 1. For D), E), and F), the initial condition is (*m*, *v*) = (0, 0).

Thus the rectangle with vertices (±m*, ±fm*) serves as an outer boundary confining all trajectories that start inside it, leading to the existence of a limit cycle (Strogatz, 2014). This result, along with the instability of the (*m*, *v*) = (0, 0) state as *fη*_*m*_ surpasses *η*_*v*_ + *k*, implies a supercritical Hopf bifurcation at *fη*_*m*_ = *η*_*v*_ + *k*. Psychologically, this means that the expectation and mood persistently oscillate under constant reality conditions, in sharp contrast to the behavior in a normal nonbipolar state (Figures 2A and 2B). It is difficult to predict how the amplitude of the oscillation scales with the psychological parameters since this requires analytically solving the nonlinear system. However, the formula for outer boundary Equation 8 could give us a prediction. Equation 8 predicts that, after the onset of bipolar disorder, the mood sensitivity *fη*_*m*_ still positively correlates with the mood amplitude. This prediction is verified by numerical calculations using large *fη*_*m*_ (Figure 2C), suggesting that the mood sensitivity parameter plays an essential role even after the onset of bipolar disorder. How the amplitude of the oscillations depends on the mood sensitivity *fη*_*m*_ ≳ *η*_*v*_ + *k* can possibly be estimated using weakly nonlinear analysis (Bender & Orszag, 1999) of Equation 3 but will not be treated here.

While the current analysis applies only in the case of constant reality, the qualitative feature of persistent oscillations does not change even if the reality *r*(*t*) varies in time. Numerical solutions show that the oscillations are not destroyed by changes in reality but take on an autonomous nature (Figures 2D and 2E). The QIDS score for the bipolar case shows intermittent peaks that match qualitatively with experimental data (Figure 2F) (Bonsall et al., 2015; Bopp et al., 2010). Together, our analyses and numerical solutions show the onset of bipolar disorder as the mood sensitivity *fη*_*m*_ crosses a critical value, leading to persistent oscillations in mood and expectation qualitatively similar to those observed in mood profiles of bipolar patients.

### Asymmetric Mood Sensitivity to Positive and Negative Events Can Lead to Unipolar Depression/Mania

**Figure 3.**
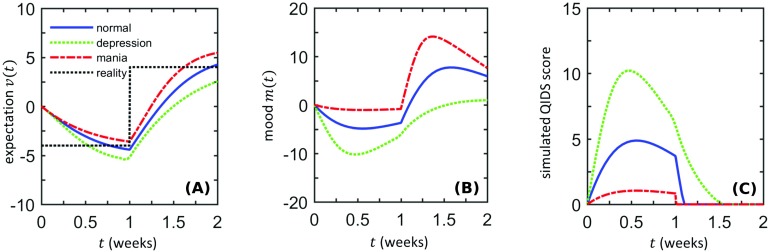
**Response to jumps in reality with *r*(*t*) = −4 for *t* ∈ [0, 1) and *r*(*t*) = 4 for *t* ∈ [1, 2].** Here normal, manic, and depressive subjects are defined by asymmetric learning rates such that (*fη*_*m*_^+^/(*η*_*v*_ + *k*), *fη*_*m*_^−^/(*η*_*v*_ + *k*)) = (0.4, 0.4), (0.8, 0.1) and (0.1, 0.8), respectively. Numerical values for other parameters, common to all subjects, are *η*_*v*_ = 1.85, *f* = 0.3, *k* = 1.85, and *k*_3_ = 0.014. Initial conditions are set to (*m*, *v*) = (0, 0). A) The predicted expectations *v* of a normal subject (solid blue), a manic subject (red dash-dotted), and a depressive subject (green dotted) all attempt to follow reality (black dotted). In the depressive state, *v*(*t*) overshoots decreases in *r*(*t*), whereas expectations in the manic state overshoot rises in *r*(*t*). B) Mood levels *m*(*t*) exhibit significant systematic differences in the normal, manic, and depressive cases, showing how asymmetric mood sensitivity can lead to unipolar depression/mania when reality *r*(*t*) is changing. C) Prolonged periods of negative mood are reflected by longer periods of large QIDS scores in depressed subjects.

Asymmetric response to positive and negative events and its effects on human learning have been widely reported and inferred from psychological experiments (Leppänen, 2006; Pulcu & Browning, 2017). It has been observed that patients with major depression respond more strongly to negative stimuli than to positive stimuli (Gotlib, Kasch et al., 2004; Gotlib, Krasnoperova et al., 2004). Patients with mania, on the other hand, show less response to negative stimuli (Lennox, Jacob, Calder, Lupson, & Bullmore, 2004). Interestingly, patients with bipolar disorder, even during euthymic or depressive episodes, show stronger responses to both positive and negative stimuli (Lawrence et al., 2004), consistent with our results in the previous subsection, where the response was characterized by the mood sensitivity *fη*_*m*_. When the learning rate for mood ηm is asymmetric (as in Equation 4) and *r* is constant, the *v* − *m* plane is split into two half-planes, separated by the nullcline $\frac{dv}{dt}=0$ (*v* = *fm* for *r* = 0). The different values *η*_*m*_^±^ apply in each of the half-planes, leading to a continuous but nondifferentiable vector field. This feature complicates the linear stability analysis (see the next sub section), but it is clear that if both *fη*_*m*_^+^ < *η*_*v*_ + *k* and *fη*_*m*_^−^ < *η*_*v*_ + *k*, the origin is linearly stable (the nonbipolar state). We show that our asymmetric learning model, even in a nonbipolar (stable when *r* is constant) state, can support unipolar depression/mania when the reality *r*(*t*) varies in time about *r* = 0. Consider three different systems with different values of *η*_*m*_^±^ such that (*fη*_*m*_^+^/(*η*_*v*_ + *k*), *fη*_*m*_^−^/(*η*_*v*_ + *k*)) = (0.4, 0.4), (0.8, 0.1) and (0.1, 0.8). These sets of learning rates will correspond to “normal,” “manic,” and “depressive” subjects, respectively. Figure 3 shows simulations started well in the past with *r*(*t* < 0) = 0. The reality is then decreased to *r*(0 ≤ *t* < 1) = −4, followed by an increase to *r*(*t* ≥ 1) = +4. We see that the expectation of depressive subjects overreacts to negative reality and fails to fully recover by *t* = 2 after the reality switched positive at *t* = 1 (Figure 3A). This lag in recovery leads to a prolonged time of depression compared to that of normal and manic subjects, reflected in both mood and QIDS scores (Figures 3B and 3C). The deviation in mood observed in the model can be explained in terms of psychology. Systems with a higher mood sensitivity for negative events will experience a larger change in mood during negative events, resulting in a lower expectation than reality. Since reality is typically changing, this overshoot in mood and expectation can last until the next event. Since systems in depressive states will always overshoot in response to negative events and undershoot in response to positive ones, their overall mood level remains lower than that of a normal system. A similar reasoning applies to subjects in a manic state, which results in average mood values higher than those in normal subjects. Interestingly, our mechanism for unipolar depression/mania is distinct from another model based on the interaction of mood and expectation (Eldar et al., 2016), which asserts that asymmetric learning rates lead to expectations higher than reality for people with depression, resulting in constant negative surprise and low mood level. Our simulations show that a different mechanism—the experimentally observed asymmetric mood sensitivity—can possibly underlie unipolar depression/mania when reality fluctuates. A rigorous analysis of the systematic deviation of mood or expectation under more general random reality functions would require more involved stochastic analysis.

### Unidirectional Changes in Asymmetric Mood Sensitivity Can Trigger Bipolar Disorder

Mathematically, bipolar disorder reveals itself in the form of a limit cycle as the origin (*m*, *v*) = (0, 0) becomes linearly unstable. When asymmetric mood sensitivity is considered, stability depends on two parameters, *fη*_*m*_^+^ and *fη*_*m*_^−^, and its delineation is more involved. Nonetheless, it is easy to show that for *r* = 0, the origin (*m*, *v*) = (0, 0) remains stable if both positive and negative mood sensitivities are below the critical value, that is, *fη*_*m*_^+^, *fη*_*m*_^−^ < *η*_*v*_ + *k*. Similarly, the origin is unstable if both mood sensitivities are above the critical value. However, when only one of them is above the critical value, the dynamics will be unstable in one half-plane defined by $\frac{dv}{dt}=0$ (*v* = *fm*) and stable in the other. In such cases, solution trajectories starting in the unstable half-plane may cross into the stable half-plane and eventually arrive at the origin. Alternatively, they may cross back into the unstable half-plane and ultimately move farther from the origin. To obtain heuristic criteria on overall system stability, we can track the trajectories of the linearized system as it traverses the two half-planes.

**Figure 4.**
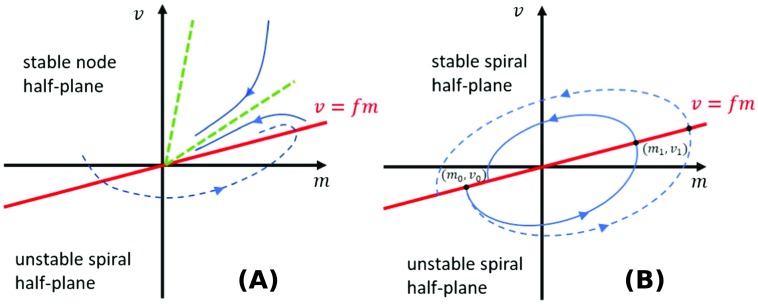
**Phase plane diagrams depicting possible scenarios of linear stability and instability.** A) Linearized dynamics in the *fm* < *v* half-plane show stable node behavior, whereas *fm* > *v* half-plane supports spiral dynamics. The overall stability is determined by the stability property of the nodal half-plane, whether or not the trajectory crosses into an unstable spiral half-plane. In the illustrated example, the green rays show the stable eigendirections. B) Both half-planes support spiral dynamics: one stable, one unstable. The overall stability is determined by whether the trajectory starting at (*m*_0_, *v*_0_) increases or decreases in magnitude as it completes a cycle.

Consider the linearization of Equations 1 and 2 about (*m*, *v*) = (0, 0) by neglecting the −*k*_3_*m*^3^ term, and assume that one half-plane is stable and the other is not; for example, *fη*_*m*_^+^ > *η*_*v*_ + *k* and *fη*_*m*_^−^ < *η*_*v*_ + *k*. Within the positive, unstable half-plane (*fm* − *v* > 0), there are two additional parameter regimes corresponding to *η*_*v*_ + *k* < *fη*_*m*_^+^ < *η*_*v*_ + *k* + $\sqrt{4\eta_{v}k}$, where the eigenvalues are complex with a positive real part, and *fη*_*m*_^+^ > *η*_*v*_ + *k* + $\sqrt{4\eta_{v}k}$, for which both eigenvalues are real and positive. We do not consider the case at the boundary values, as it holds for only very special parameter relationships. Approaching the origin from the positive half-plane, these two parameter regimes give rise to an unstable spiral and an unstable node, respectively. Similarly, there are two regimes in the negative plane, (*fm* − *v* < 0): *η*_*v*_ + *k* > *fη*_*m*_^−^ > *η*_*v*_ + *k* − $\sqrt{4\eta_{v}k}$ and *fη*_*m*_^−^ < *η*_*v*_ + *k* − $\sqrt{4\eta_{v}k}$, corresponding to a stable spiral and a stable node at the origin, respectively. Nodes determine the stability/instability of the origin over spirals since most solutions starting in the node half-plane will stay in that half-plane. The only exceptions are trajectories starting in the wedge between the eigenvector corresponding to an eigenvalue with a larger absolute value and the half-plane boundary *v* = *fm*. As shown in Figure 4A, if a node and a spiral coexist, solutions starting in the spiral half-plane (or the wedge in the node half-plane) will end up in the part of the node half-plane and follow the stability properties of the node. Hence the node determines the stability when it coexists with a spiral.

When spiral node behavior arises in both half-planes, the two spiral dynamics alternate along the trajectory and compete in strength. As shown in Figure 4B, solutions starting in one half-plane will enter the other after half a cycle. We can deduce the overall stability by tracking trajectories through a full cycle and returning to the original half-plane. The stability can be inferred from determining the change in magnitude of the trajectory after a full cycle. In the positive half-plane, the general form of solutions is

$$m(t)=e^{\frac{f\eta_{m}^{+}-\eta_{v}-k}{2}t}(A\cos(\frac{\sqrt{4\eta_{v}k-{\left(f\eta_{m}^{+}-\eta_{v}-k\right)}^{2}}}{2}t)+B\sin(\frac{\sqrt{4\eta_{v}k-{\left(f\eta_{m}^{+}-\eta_{v}-k\right)}^{2}}}{2}t))$$ (9)

$$v(t)=e^{\frac{f\eta_{m}^{+}-\eta_{v}-k}{2}t}(C\cos(\frac{\sqrt{4\eta_{v}k-{\left(f\eta_{m}^{+}-\eta_{v}-k\right)}^{2}}}{2}t)+D\sin(\frac{\sqrt{4\eta_{v}k-{\left(f\eta_{m}^{+}-\eta_{v}-k\right)}^{2}}}{2}t)).$$ (10)

Here the constants *A*, *B*, *C*, *D* are determined by the initial conditions associated with the linearized equations but do not affect the following stability analysis. Suppose a trajectory starts on the boundary of the two half-planes at (*m*_0_, *fm*_0_). After a time of $\Delta t=\frac{2\pi }{\sqrt{4\eta_{v}k-{\left(f\eta_{m}^{+}-\eta_{v}-k\right)}^{2}}}$, the trajectory reaches the boundary again at point (*m*_1_, *fm*_1_). The distance to origin will change by a multiplicative factor of $\exp\left[\frac{\pi (f\eta_{m}^{+}-\eta_{v}-k)}{\sqrt{4\eta_{v}k-{\left(f\eta_{m}^{+}-\eta_{v}-k\right)}^{2}}}\right]$. A similar argument applies to the neg ative half-plane, and the criterion for the solution to move closer to the origin after a full cycle, that is, linear stability, is

$$\frac{\pi (f\eta_{m}^{+}-\eta_{v}-k)}{\sqrt{4\eta_{v}k-{\left(f\eta_{m}^{+}-\eta_{v}-k\right)}^{2}}}+\frac{\pi (f\eta_{m}^{-}-\eta_{v}-k)}{\sqrt{4\eta_{v}k-{\left(f\eta_{m}^{-}-\eta_{v}-k\right)}^{2}}}<0.$$ (11)

**Figure 5.**
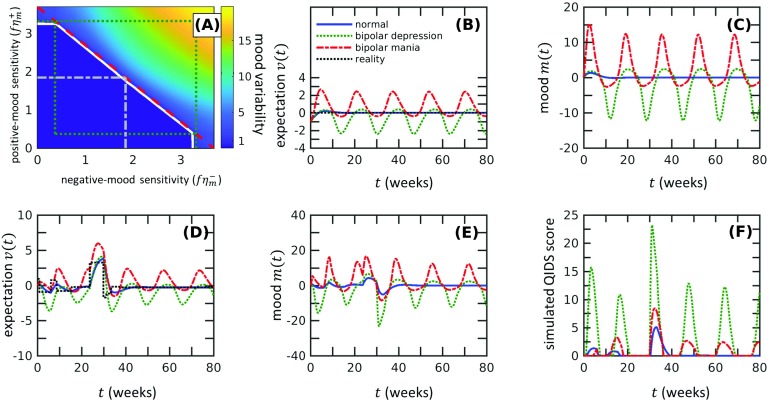
**Bipolar disorder can be triggered by large unidirectional changes in mood sensitivity, even when one of the mood sensitivities does not cross the stability threshold.** A) Numerical computations were performed within the period *t* ∈ [0, 162.5] using *r*(*t* > 0) = 0. The stability is characterized by the standard deviation of mood when *t* ∈ [81.25, 162.5], and the stability boundary (white solid curve) is determined by the contour of mood variability of a model with critical mood sensitivities, that is, *fη*_*m*_^+^ = *fη*_*m*_^−^ = *k* + *η*_*v*_. Other parameter values used in the simulations are *η*_*v*_ = 1.48, *f* = 0.3, *k* = 0.37, and *k*_3_ = 2.8 × 10^−3^. The curve *f*(*η*_*m*_^+^ + *η*_*m*_^−^) = 2(*k* + *η*_*v*_) (red-dashed line) solves Equation 11 and matches well with the numerically computed stability boundary (white solid curve) when both half-planes support spirals (inside the green-dotted box). When both half-planes are stable (inside the gray-dot-dashed box), the solutions are stable, as expected, since eigenvalues in both half-planes have negative real parts. When one half-plane is an unstable spiral and the other is a stable node (upper left and lower right rectangles with one gray-dot-dashed and two green-dotted sides), the solutions are stable according to our analysis in Figure 4, consistent with the numerical results. Finally, when an unstable node is present (upper and right to green dotted lines), the system is unstable. We show that the coexistence of stable spiral and unstable node half-planes leads to instability. Stability of the case in which both stable and unstable node half-planes arise depends on initial conditions. B) Under constant reality, bipolar disorder triggered by mood sensitivity asymmetry in different directions induces different behavior in expectation *v*(*t*). Compared to the normal state (solid blue), higher negative mood sensitivity [depressive bipolar state, *fη*_*m*_^−^ = 2(*η*_*v*_ + *k*) and *fη*_*m*_^+^ = 0.5(*η*_*v*_ + *k*)] lowers expectations (green-dotted lines), while higher positive mood sensitivity [manic bipolar state, *fη*_*m*_^−^ = 0.5(*η*_*v*_ + *k*) and *fη*_*m*_^+^ = 2(*η*_*v*_+ *k*)] leads to higher expectations (red dash-dotted). Initial conditions are (*m*, *v*) = (0, −1). Parameter values used in this and in the following subfigures are *η*_*v*_ = 0.37, *f* = 0.3, *k* = 0.37, and *k*_3_ = 2.8 × 10^−3^. C) Under constant reality, bipolar disorder induced by asymmetry in mood sensitivities in different directions biases the mood *m*(*t*) in different directions. D) The biases in the asymmetry-induced oscillations in the expectation persist under random reality conditions, with depressive/manic bipolar states leading to statistically lower/higher expectations. The realization of reality is drawn as described with *σ*_*r*_ = 2, *k*_*r*_ = 1. Initial conditions: (*m*, *v*) = (0, 0). E) The mood trajectories *m*(*t*) show qualitatively similar biases as in B). F) Predicted QIDS scores of depressive and manic bipolar individuals are often higher than those in normal individuals.

This approximate stability analysis is consistent with the results of the numerical simulations shown in Figure 5A. When spirals are induced in both half-planes, the origin is stable when *f*(*η*_*m*_^+^ + *η*_*m*_^−^) < 2(*η*_*v*_ + *k*). Since $\sqrt{4\eta_{v}k}$ − (*fη*_*m*_^+^ − *η*_*v*_ − *k*)^2^ is symmetric for *fη*_*m*_ around *η*_*v*_ + *k*, it is clear that *f*(*η*_*m*_^+^ + *η*_*m*_^−^) = 2(*η*_*v*_ + *k*) (red dashed line) leads to equality in Equation 11. This approximate analytic stability boundary (white solid line) closely matches that inferred from numerical solutions. Coexistence of a node and a spiral yields the same stability as that of the node half-plane, predicted by our analysis. However, when a stable node coexists with an unstable node, numerical solutions show that the origin is always unstable. The simple linear analysis does not establish the existence of a limit cycle in this case, so the instability may not correspond to bipolar disorder.

Bipolar disorders triggered by asymmetric mood sensitivities show oscillation in mood and expectation that are similar to those predicted in the symmetric case, but they contain systematic biases (Figures 5B and 5C) that were not observed in the symmetric case. As in unipolar depression/mania, the biases in mood and expectation always have the same sign; that is, mood and expectations are systematically either both lower or higher. The depression-biased case may describe Type II bipolar disorder. The same pattern persists when the reality is treated as random (Figures 5D and 5E), with the mood and expectation responding to changes in reality as well as exhibiting their intrinsic oscillations. As expected, the predicted QIDS scores for depressive bipolar (or Type II) subjects are much higher than those of normal and manic bipolar subjects, but even bipolar manic subjects are predicted to exhibit larger QIDS scores than normal individuals (Figure 5F). Moreover, manic and depressive bipolar subjects could often show high QIDS scores when normal individuals have stable moods. Our numerics suggest that bipolar disorder can be caused by extreme asymmetry in mood sensitivity, which leads to systematically biased mood and expectation patterns. The direction and magnitude of mood sensitivity asymmetry may be an underlying feature of different types of asymmetric bipolar disorders.

### Effects of Antidepressants and Lithium

**Figure 6.**
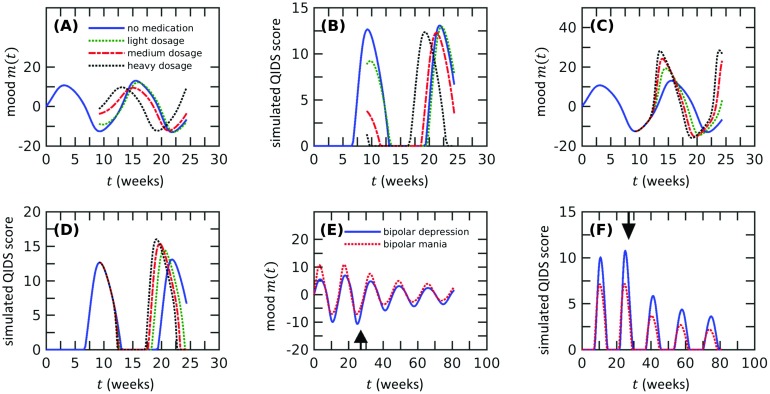
**Possible effects of antidepressants and lithium on subjects with bipolar disorder, includ ing the mania-inducing effect of antidepressants and the sedative effects of lithium, are assessed in our model.** A) Numerical calculation of the mood of a bipolar subject (solid blue curve) using *fη*_*m*_ = 1.5(*η*_*v*_ + *k*). At *t* = 9.2 weeks, within a depressive episode, the patient is treated with antidepressants, modeled by an elevation in mood (Goldbeter, 2011). Trajectories corresponding to dosages that instantaneously decrease the depression to 70% of its lowest value (green dotted), 30% of its lowest value (red dash-dotted), and 10% of its lowest value (black dotted) are shown. Note that higher doses lead to an earlier onset of mania. This antidepressant-induced mania is observed clinically (Altshuler et al., 1995; Goldberg & Truman, 2003). The numerical values for the simulations are *η*_*v*_ = 0.37, *f* = 0.3, *k* = 0.37, and *k*_3_ = 2.8 × 10^−3^; the initial conditions are (*m*, *v*) = (0, −1). B) The quick transition to a manic phase results in a depressive episode that occurs sooner than in untreated subjects, as indicated by an earlier peak in QIDS score for subjects treated with a high antidepressant dose. C) When the effect of antidepressants is modeled by an increased positive mood sensitivity, an earlier manic episode is observed with larger amplitude. The frequency of mood oscillation also increases as dosage increases. The positive mood sensitivities used in the simulations for low to high dosage are *fη*_*m*_^+^ = 2.25(*η*_*v*_ + *k*), 3(*η*_*v*_ + *k*), 3.75(*η*_*v*_ + *k*), respectively, while the negative mood sensitivities are the same as those used in A). D) The quick transition to mania also induces an earlier depressive episode, with larger QIDS score as the dosage increases. E) Simulated mood dynamics for mania-biased mood sensitivity asymmetry [red dotted, *fη*_*m*_^+^ = 1.5(*η*_*v*_ + *k*), *fη*_*m*_^−^ = (*η*_*v*_ + *k*)] and depression-biased mood sensitivity asymmetry [blue solid, *fη*_*m*_^+^ = (*η*_*v*_ + *k*), *fη*_*m*_^−^ = 1.5(*η*_*v*_ + *k*)]. The sedative effects of lithium are modeled via a symmetric 20% reduction in mood sensitivity and are implemented in our numerics at *t* = 27.1 weeks (black arrow). This treatment decreases oscillation amplitudes consistent with clinical observations (Phiel & Klein, 2001). F) The reduction in mood oscillation amplitudes yields smaller predicted QIDS scores.

In this section, we explore the effects of common medications used to treat bipolar disorder. First, we want to see if our model can explain the antidepressant-induced mania seen in bipolar patients. Antidepressants are a category of medicine for treating depression disorder, and their effects on patients with depression are significant (Morris & Beck, 1974). For patients with bipolar disorder, it has been reported that 20%–40% of their manic episodes are induced by antidepressants (Altshuler et al., 1995; Goldberg & Truman, 2003). This unanticipated effect was previously studied by Goldbeter (2011) using a bistability model of depression and mania. Our model for bipolar disorder is intrinsically oscillatory, and it is not clear whether there is a threshold of dosage above which the manic episodes will be induced, as predicted in Goldbeter (2011). Nevertheless, when the effect of antidepressants is modeled by a shift in mood (Goldbeter, 2011), simulations of our model show that there is, indeed, a threshold of dosage below which a transient alleviation of depression occurs, followed by a usual manic episode. Above this dose threshold, manic episodes are induced earlier (Figure 6A). This result is surprising since small perturbations in mood do not qualitatively change the subsequent dynamics and our model does not have a built-in mechanism for bistability. Subjects treated with high doses of antidepressants are predicted to show a phase shift in the mood oscillations (Figure 6A). This phase shift would yield an earlier peak in the QIDS score (Figure 6B). Another way to model the effect of antidepressants is to increase the positive mood sensitivity (Harmer, 2008). This effect also leads to an earlier manic episode, but with greater strength and a higher frequency (Figure 6C). The QIDS score also shows a sooner and stronger depressive episode (Figure 6D). The observed rapid cycle is consistent with clinical observations (Altshuler et al., 1995).

The sedative effects of lithium were first discovered in 1949, but its molecular mechanisms of action have not yet been fully elucidated (Corbella & Vieta, 2003; Phiel & Klein, 2001). Nonetheless, lithium is one of the most prescribed treatments for bipolar disorder (Phiel & Klein, 2001). While our model does not explicitly involve details at the molecular level, it suggests a crucial behavioral property, characterized by the mood sensitivity, that might be regulated by lithium. To see this, we simulate the mood *m*(*t*) in the bipolar state and decrease the mood sensitivity parameter after a certain time point (Figure 6E). We observe that after the mood sensitivity is decreased, the amplitudes of oscillations in mood gradually decrease, eventually becoming constant over time. Depression is lessened after treatment, as indicated by a decrease in the QIDS score (Figure 6F); moreover, decreases in mood sensitivity do not induce mania. In contrast to antidepressants, lithium does not trigger manic episodes, which makes it suitable to treat bipolar depression (Phiel & Klein, 2001). This result suggests that the sedative effect of lithium might be achieved by decreasing the mood sensitivity parameter rather than directly modifying mood.

## DISCUSSION AND CONCLUSION

Existing models for bipolar disorder are based on one of two basic mechanisms: bistability and biological rhythm. Models invoking bistability assume that there are multiple stable states representing different phenotypes of depression and mania. Here variations in mood are triggered by random external perturbations arising from life events (Cochran et al., 2017; Steinacher & Wright, 2013). Biological rhythms assume an intrinsic oscillation in the brain. In this case, mood oscillations persist without perturbations (Bonsall et al., 2015; Daugherty et al., 2009; Eldar & Niv, 2015; Goldbeter, 2011; Mason et al., 2017). In this work, we proposed and analyzed a variant of a model by Eldar et al. (2016) for bipolar disorder based on the intrinsically oscillating interaction between mood and expectation. Our model exhibits oscillatory mood behavior when the mood sensitivity exceeds a threshold. Previous models have explained such oscillations via the dynamics of intrinsic brain circuits or mutual inhibition of depression and mania (Bonsall et al., 2015; Daugherty et al., 2009; Goldbeter, 2011). Our model proposes that mood oscillations arise from a psychological mechanism in which high expectation induces high mood until it reaches a physiological limit. The mood then decreases, followed by a concomitant decrease in expectation. This mechanism is similar to that proposed by Eldar and Niv (2015), Eldar et al. (2016), and Mason et al. (2017), but we identified a key psychological property, defined by the mood sensitivity *fη*_*m*_^±^, that may control a whole spectrum of states, from normal to cyclothymic personality to Type I and Type II bipolar disorders. Measuring mood sensitivity may result in a more refined method to diagnose, classify, and describe such disorders.

The perturbations from life events in biological rhythm models are usually treated as a noise term in oscillator models. We have modeled life events explicitly by a known time-dependent reality function *r*(*t*) to explore the response of our model to specific changes in reality. This also enables a direct comparison of the two mechanisms since different forms of *r*(*t*) can be used to investigate which mechanism better explains the observations. For example, when an individual experiences a prolonged negative life event, biological rhythm models would predict a persistence in mood oscillation, while bistability models would likely predict a prolonged state of depression. By directly incorporating reality *r*(*t*) into models with different central mechanisms and then comparing their predictions with observations, we may be able to decide which model better describes bipolar disorder. This may also reveal a need for the combination of different mechanisms.

We also explored in detail the effects of asymmetric mood sensitivity on unipolar depression/mania and bipolar disorder. Humans are known to react differently toward positive and negative events (Pulcu & Browning, 2017), and patients with major depression and bipolar disorder have a stronger bias toward these events (Leppänen, 2006). It has been suggested by Eldar et al. (2016) that this asymmetry can lead to unrealistic expectation and low mood in depressive patients. Our analysis shows that depression can result from a higher mood sensitivity toward negative events, which leads to a reasonable expectation but negative mood. Our model also predicts that depression is a dynamical phenomenon; that is, when no strong environmental stimulus is present, depressive patients may appear normal, but they react more negatively than normal subjects once reality fluctuates. Our prediction is supported by clinically observed processing bias (Fu et al., 2004), but additional psychological experiments should be performed to test our model hypotheses. Our model also shows that unidirectional changes in mood sensitivity can trigger a full bipolar state. Our mathematical framework can explain the paradoxical observation that while depressive patients react more strongly to negative events, bipolar patients in the depressive phase can react more strongly to positive events (Lawrence et al., 2004; Leppänen, 2006). Asymmetry in the mood sensitivity introduces an interesting mathematical question on stability. Conventionally, the local stability of an equilibrium is determined by the stability of the system linearized around the equilibrium point (Strogatz, 2014). To analyze our model with an asymmetric parameter, we concatenated the linear solutions in the two half-planes. Our conclusions accurately match those derived from numerical simulations of our full nonlinear model.

Our work focused on the effect of mood sensitivities on unipolar depression/mania and bipolar disorder. Similar analyses can be carried out with an emphasis on, for example, the expectation learning rate *η*_*v*_ or linear decay rate of mood *k*, but clarification of the main parameter that triggers bipolar disorder would require experimental input, such as quantification of those parameters from both normal and bipolar subjects. The characterization of mania can include complex, multidimensional traits, such as irritability, rapidity of thoughts, inability to concentrate, or increased goal-directed behavior (Bauer et al., 1991). Despite this, models for bipolar disorder, including ours, simplify mania to a one-dimensional variable to focus on the bipolar behavior of mood (Bonsall et al., 2015; Eldar & Niv, 2015; Goldbeter, 2011). Development and analysis of multidimensional models, such as a goal-directed behavior BAS-type model, may highlight the role of more specific traits in triggering bipolar disorder (Urošević et al., 2008).

Owing to a lack of understanding of the underlying physiological mechanisms of bipolar disorder, the parameters in models, including ours, for bipolar disorder are often phenomenological and treated as fitting parameters to the experimental data. However, we have identified parameters that can be expressed in psychological terms, such as learning rate for expectation or recovery rate for mood, that can be measured by psychological experiments instead of fitting to data. For example, reaction toward events can be measured by fMRI or pupilometry (Fu et al., 2004; Lawrence et al., 2004; Pulcu & Browning, 2017), which can then be used to estimate the learning rates and the mood sensitivity parameter. In fact, the measurements of Lawrence et al. (2004) showing that bipolar patients react more strongly to both positive and negative events agree with our model predictions.

Finally, our model parameters have been assumed to be constant in time. In reality, higher order nonlinearities may arise if these physiological parameters themselves depend on mood and expectation. At the cellular level, neural synapses can be modified by the synaptic current (Fain, 1999), which suggests that recurrence of negative events might strengthen reactions to them. It has been observed that depression is correlated to chronic pain (Geisser, Roth, Theisen, Robinson, & Riley III, 2000) and that an initial depression might become long term because of prolonged negative realities like environmental difficulties and lack of social support, the so-called cognitive vulnerability (Persons & Miranda, 1992). This evidence suggests a possibility that the psychological parameters in our model are dynamical and affected by the environment instead of heredity, so the depression persists even if the reality returns to normal level. Therefore a natural next step in our work is to incorporate the dynamics of mood sensitivity as well as other parameters—that incidentally may lead to bistability—to explore how recurrences of external events can trigger depression/mania or bipolar disorder.

## AUTHOR CONTRIBUTION

Shyr-Shea Chang: Conceptualization: Equal; Formal analysis: Lead; Investigation: Lead; Visualization: Lead; Writing – original draft: Lead; Writing – review & editing: Equal. Tom Chou: Conceptualization: Equal; Funding acquisition: Lead; Supervision: Lead; Writing – review & editing: Equal.

## FUNDING INFORMATION

The authors are grateful for support from the Army Research Office (W911NF-14-1-0472 and W911NF-18-1-0345) and the National Science Foundation (DMS-1516675 and DMS-1814364). SSC was supported in part by the Systems and Integrative Biology predoctoral training grant (T32GM008185).
